# Transportation Noise and Blood Pressure in a Population-Based Sample of Adults

**DOI:** 10.1289/ehp.1103448

**Published:** 2011-09-01

**Authors:** Julia Dratva, Harish C. Phuleria, Maria Foraster, Jean-Michel Gaspoz, Dirk Keidel, Nino Künzli, L.-J. Sally Liu, Marco Pons, Elisabeth Zemp, Margaret W Gerbase, Christian Schindler

**Affiliations:** 1 Department of Epidemiology and Public Health, Swiss Tropical and Public Health Institute, Basel, Switzerland; 2 University of Basel, Basel, Switzerland; 3 Centre for Research in Environmental Epidemiology, Barcelona, Spain; 4 Municipal Institute of Medical Research–Hospital del Mar, Barcelona, Spain; 5 Universitat Pompeu Fabra, Barcelona, Spain; 6 Centro de Investigación Biomédica en Red (CIBER) Epidemiologia y Salud Pública, Barcelona, Spain; 7 Department of Community Medicine and Primary Care, University Hospitals, Geneva, Switzerland; 8 Department of Environmental and Occupational Health Sciences, University of Washington, Seattle, Washington, USA; 9 Ospedale Regionale di Lugano, Lugano, Switzerland; 10 Division of Pulmonary Medicine, University Hospitals, Geneva, Switzerland

**Keywords:** blood pressure, diabetes, epidemiology, hypertension, noise

## Abstract

Background: There is some evidence for an association between traffic noise and ischemic heart disease; however, associations with blood pressure have been inconsistent, and little is known about health effects of railway noise.

Objectives: We aimed to investigate the effects of railway and traffic noise exposure on blood pressure; a secondary aim was to address potentially susceptible subpopulations.

Methods: We performed adjusted linear regression analyses using data from 6,450 participants of the second survey of the Swiss Study on Air Pollution and Lung Disease in Adults (SAPALDIA 2) to estimate the associations of daytime and nighttime railway and traffic noise (A-weighted decibels) with systolic blood pressure (SBP) and diastolic blood pressure (DBP; millimeters of mercury). Noise data were provided by the Federal Office for the Environment. Stratified analyses by self-reported hypertension, cardiovascular disease (CVD), and diabetes were performed.

Results: Mean noise exposure during the day and night was 51 dB(A) and 39 dB(A) for traffic noise, respectively, and 19 dB(A) and 17 dB(A) for railway noise. Adjusted regression models yielded significant effect estimates for a 10 dB(A) increase in railway noise during the night [SBP β = 0.84; 95% confidence interval (CI): 0.22, 1.46; DBP β = 0.44; 95% CI: 0.06, 0.81] and day (SBP β = 0.60; 95% CI: 0.07, 1.13). Additional adjustment for nitrogen dioxide left effect estimates almost unchanged. Stronger associations were estimated for participants with chronic disease. Significant associations with traffic noise were seen only among participants with diabetes.

Conclusion: We found evidence of an adverse effect of railway noise on blood pressure in this cohort population. Traffic noise was associated with higher blood pressure only in diabetics, possibly due to low exposure levels. The study results imply more severe health effects by transportation noise in vulnerable populations, such as adults with hypertension, diabetes, or CVD.

Noise pollution is an increasing problem in our modern society (Ouis 2002). Apart from aircraft noise, traffic noise is perceived as the dominant source of noise, but other transportation-related sources of noise are also increasing. Common disturbances of noise are interference of communication, concentration, and sleep (Griefahn et al. 2000; Ising and Kruppa 2004). An increasing body of literature has shown traffic noise to have adverse short- and long-term health effects (Babisch 2006; Berglund et al. 1999; Bluhm et al. 2007; Stansfeld et al. 2000, 2005). One of the suggested mechanisms by which noise affects nonauditory health is through indirect or direct activation of the sympathetic nervous system and endocrine systems (Ising and Kruppa 2004; Stansfeld and Matheson 2003), resulting in autonomic reactions, including increased blood pressure, heart rate, and arrhythmia (Berglund et al. 1999). Therefore, research has focused on the impact of transportation noise on cardiovascular health. There is suggestive evidence that transportation noise exposure is associated with an increase in ischemic heart disease (Babisch 2006). Associations between traffic noise and hypertension have been inconsistent (Babisch et al. 2006; Chang et al. 2009; Jarup et al. 2008; van Kempen et al. 2006), whereas recent studies on aircraft noise have shown positive associations (Babisch and Kamp 2009; Haralabidis et al. 2010).

Most previous studies have investigated exposure to traffic noise, the most common noise pollutant, and aircraft noise, the loudest exposure. However, in densely populated countries with a dense railway network, such as Switzerland, railway noise should also be addressed. The Swiss Federal Office for the Environment (FOEN) calculated that an area of 35 km^2^ in daytime and 56 km^2^ in nighttime is exposed to noise levels above accepted thresholds; 100% of this area is located in urban and highly populated areas, and nighttime train traffic is increasing, partly due to an increase of freight train traffic at night [Bundesamt für Umwelt (BAFU) 2009a]. In Switzerland, trains are electrically powered, whereas trucks and cars are usually powered by diesel engines. Our primary aim was to investigate the long-term association of railway noise and traffic noise exposure with blood pressure in the adult Swiss Study on Air Pollution and Lung Disease in Adults (SAPALDIA) cohort, to gain further knowledge on the cardiovascular impact of transportation noise exposure in the general population and potentially susceptible subpopulations. The SAPALDIA data on air pollution exposure at the household level further enabled an adjustment for air pollution indicators.

## Materials and Methods

The study sample for this analysis includes 6,450 men and women, 28–72 years of age, from the second survey of the SAPALDIA cohort study in 2002/2003, with objective measures on blood pressure. The SAPALDIA cohort has been described in detail elsewhere (Ackermann-Liebrich et al. 2005). In short, the SAPALDIA study population was recruited in 1991 as a population-based, random sample of adults (18–60 years of age) from eight study areas in Switzerland, representing a broad range of environmental conditions (Basel, *n* = 799; Wald, *n* = 1,179; Davos, *n* = 540; Lugano, *n* = 929; Montana, *n* = 616; Payerne, *n* = Aarau, *n* = 989; Geneva, *n* = 551). The second survey held in 2002/2003 repeated the main clinical measurements, lung function, and cardiovascular health measures, as well as the main questionnaire, including questions on sociodemographic characteristics, lifestyle factors, living, housing and work-related characteristics, and health status.

*Exposure assessment.* Information on the exposures of interest, average railway and traffic noise during day (0600 hr to 2200 hours) and night (2200 hr to 0600 hours), was obtained from SONBASE, the national databank on noise pollution in Switzerland, developed by the FOEN (BAFU 2009a, 2009b). The comprehensive noise calculations incorporated input data from the federal offices for spatial development, roads, transport, civil aviation, statistics and civil protection, and sport. Noise propagation was modeled using the STL86+ emission model (BAFU 2009b) for traffic noise and the SEMIBEL (Schweizerisches Emissions- und Immissionsmodell für die Berechnung von Eisenbahnlärm) for railway noise. Noise levels, expressed as rated sound levels [A-weighted decibels; dB(A)], were calculated for 10 × 10 m grids within the geographic area, as well as for individual buildings. The SONBASE noise model assumes that only households within a 1,000-m radius of railway tracks experience railway noise; households outside this radius are assigned a value of 0 dB(A) for railway noise. An indicator variable was included in the model to indicate the particpants with no railway exposure estimate. For the present analyses, the SONBASE data on railway and traffic noise were linked to SAPALDIA participants’ home addresses. The reported standard deviation for railway noise is lower than that for traffic noise [2.0 dB(A) and 2.6 dB(A), respectively] (BAFU 2009b). For the basic model for Switzerland as a whole, a historical validation was performed that indicated good agreement for noise levels ≥ 60 dB(A). In addition, the model for traffic noise showed reasonable agreement with local models from three Swiss cantons with more detailed input data [mean ± SD difference –0.6 ± 1.9 dB(A)] (BAFU 2009b).

Annual average air pollution exposures for outdoor particulate matter (PM) ≤ 10 μm in aerodynamic diameter (PM_10_) at each participant’s residence in the year before the blood pressure measurement was predicted by a dispersion model (Liu et al. 2007), and nitrogen dioxide (NO_2_) estimates were calculated using a hybrid model (Liu et al. 2012).

*Outcome of interest.* Systolic blood pressure (SBP) and diastolic blood pressure (DBP) were measured by the Riva-Rocci method (in millimeters of mercury) at the SAPALDIA study centers by trained fieldworkers after at least 10 min of rest in a seated position, using an automatic OMRON 705 CP (OMRON, Tokyo, Japan) with the cuff attached to the naked left upper arm (Ackermann-Liebrich et al. 2005). Two measurements taken 3 min apart were averaged to obtain SBP and DBP values used in the analysis.

*Covariates.* Variables considered as potential confounders or effect modifiers were self-reported physician-diagnosed chronic diseases (hypertension, heart attack, stroke, diabetes, kidney disease, hearing deficit), antihypertensive treatment (calcium antagonists, angiotensin-converting enzyme blockers, diuretics, and beta blockers), general health indicators [smoking, physical activity, and body mass index (BMI)], sociodemographic characteristics (age, education, employment status), self-reported work-related noise exposure, and housing characteristics (type of home, double-glass windows, number of persons living in the same home, years of residency, and construction year of building). Participants also indicated their level of traffic noise annoyance at home with open windows on an 11-point scale, which was evaluated as a potential effect modifier.

*Statistical analyses.* First, we evaluated the distribution of various characteristics of the entire sample and for subsamples with and without self-reported physician-diagnosed hypertension. We calculated the prevalence of self-reported physician-diagnosed hypertension and the prevalence of hypertension based on self-reported hypertension, antihypertensive medication use, or blood pressure measurements indicating SBP > 140 mmHg and DBP > 90 mmHg (Pickering et al. 2005). The association of transportation noise exposure and cross-sectional hypertension was assessed by logistic regression. Pearson’s correlation coefficients between railway noise, traffic noise, and home outdoor estimates of PM_10_ were calculated.

Second, we used mixed linear regression models with random intercepts for the different study areas to estimate associations of daytime and nighttime traffic and railway noise exposure with blood pressure. Potential covariates were selected based on univariate associations and biological plausibility and retained in final models based on a backward selection process with *p* < 0.20. The final model included sex, age, age as quadratic polynomial; marital status; educational status (low, medium, high); working status (full time/part time); occupational noise exposure (yes/no); smoking status (never, former, current smoker); alcohol consumption (daily, weekly, seldom/never); use of antihypertensive medication in last month; physician diagnosed hypertension, diabetes, and hearing deficit; and objective measures of mean pulse, BMI, cholesterol, and estimated home outdoor exposure to NO_2_. Study area was included as a random effect variable. We estimated effects based on a series of models of increasing complexity to assess the influence of different sets of confounders. The first model included sociodemographic variables only; the second added life-style factors previously associated with hypertension; the third also included medical history characteristics potentially associated with noise, hypertension, or both; the fourth added self-reported exposure to noise at work; and the fifth and final model included NO_2_, or (as a sensitivity analysis) PM_10_ instead of NO_2_.

To investigate differential susceptibility to noise exposure in subpopulations, we stratified analyses by sex, self-reported physician-diagnosed hypertension, cardiovascular disease (CVD; reported myocardial infarction or stroke) and diabetes; high versus low level of noise annoyance at home; high versus low risk of noise misclassification; and residential stability. Low misclassification was assumed to be low for participants who were neither employed full time, and therefore presumably spent more time at home, nor exposed to occupational noise. Residential stability was based on the question “Do you live in the same home as when you were last surveyed?” Participants who had not moved since the last survey were considered “stable.”

We ran further sensitivity analyses to estimate the potential bias introduced by missing railway estimates for 1,651 participants living within the 1,000-m corridor who were missing data for railway noise and for the 1,392 subjects who lived > 1,000 m from the railway lines classified as having no railway noise exposure. First, we assigned those participants without railway noise data a value equal to the 10th percentile or 25th percentile of daytime or nighttime railway noise exposure among those with estimated values and estimated the associations between daytime and nighttime railway noise and blood pressure. In addition, we estimated associations after excluding all subjects with missing railway estimates, or excluding subjects with missing data living within the 1,000-m corridor only. Missing values in traffic noise were negligible (*n* = 75).

All analyses were done using the STATA statistical software package (version 10; Stata Corporation, College Station, TX, USA). Statistical significance was assumed at a level of *p* < 0.05.

The study protocol was conducted according to Swiss and international ethical regulations and reviewed by the Swiss cantonal ethical commissions of the respective study areas. Study participants gave written, informed consent before study participation.

## Results

The mean age of the study participants was 52 years, and approximately half were female (Table 1). Self-reported physician-diagnosed hypertension was reported by 17% of the study population (men, 17.6%; women, 14.7%; *p* = 0.002). When hypertension was defined as self-reported doctor-diagnosed hypertension, antihypertensive medication use, or elevated blood pressure measurements, the prevalence of hypertension was 37%. No significant association was seen between transportation noise and prevalence of hypertension, either for self-reported hypertension or the combined hypertension definition (data not shown). With the exception of mean pulse, self-reported occupational noise exposure, and noise annoyance, hypertensive and nonhypertensive participants differed significantly with regard to the predictors and confounders considered in this analyses (Table 1). Twenty-eight percent of the study population was exposed to daytime traffic noise above the maximum noise levels allowed in living quarters [≥ 55 dB(A)], and 19% to higher nighttime traffic noise [≥ 45 dB(A)]; 1.5% were exposed to higher railway noise at day, and 1% at night [≥ 45 dB(A)] (BAFU 2009a). Noise exposure was largely heterogeneous across study centers. The data yielded higher noise exposures in urban study areas (data not shown). Exposure levels by day and night correlated highly within each specific noise source (*r* = 0.90 for railway noise and 0.98 for traffic noise; Table 2). Correlation between air pollution indicators NO_2_ and PM_10_ and transportation noise was generally low, ranging from *r* = 0.16 for PM_10_ and daytime traffic noise to *r* = 0.37 for PM_10_ and nighttime railway noise (Table 2). Correlation of traffic noise with NO_2_ was higher than with PM_10_ in rural centers, whereas in urban study centers, this was not the case (data not shown).

**Table 1:** Characteristics of the study population as a whole and of the subsamples of subjects with and without self-reported physician-diagnosed hypertension.*a*

Total study (*n* = 6,450)	Stratum by hypertension*a*		
	Characteristic	No (*n* = 5,336)	Yes (*n* = 1,073)	*p*-Value*b*
Sociodemographic characteristics								
Sex (%)								
Male		48.9		47.8		54.1		
Female		51.1		52.3		46.0		< 0.0001
Age (mean ± SD)		52.0 ± 11.5		50.5 ± 11.3		60.3 ± 8.5		< 0.0001
Marital status (%)								
Married/widowed		74.0		72.6		80.9		
Divorced		9.0		9.2		8.5		
Single		16.0		17.2		10.1		< 0.0001
Educational level*c* (%)								
Low		22.8		21.7		28.7		
Medium		59.6		60.4		55.3		
High		17.6		17.9		15.8		< 0.0001
Full-time employment (%)		48.0		50.6		34.2		< 0.0001
Lifestyle characteristics								
Smoking (%)								
Never		43.4		44.4		38.8		< 0.0001
Former		31.6		30.0		39.7		
Current		24.9		25.5		21.5		
BMI (mean ± SD)		25.9 ± 4.4		25.3 ± 4.1		28.7 ± 4.6		< 0.0001
Alcohol use (%)								
Seldom/never		30.8		30.8		30.8		
Weekly		44.6		46.3		35.6		
Daily		24.7		22.9		33.6		< 0.0001
Health status characteristics								
Blood pressure (mmHg) (mean ± SD)								
SBP		127 ± 19.5		123 ± 18		142 ± 20		< 0.0001
DBP		80 ± 11.0		78 ± 10.5		89.9 ± 11		< 0.0001
Antihypertensive medication (%)		11.3		4.0		47.7		< 0.0001
Pulse [counts/min (mean ± SD)]		70 ± 10.5		70 ± 10		71 ± 12		0.273
Cholesterol [mmol/L (mean ± SD)]		6.03 ± 1.3		6.01 ± 1.2		6.12 ± 1.5		0.008
Diabetes*a* (%)		3.0		1.6		10.0		< 0.0001
CVD*a* (%)		7.7		53		19.9		< 0.0001
Hearing deficit*a* (%)		5.8		4.9		10.3		< 0.0001
Self-reported noise exposure								
Noise exposure at work*d* (%)		13.9		13.9		14.3		0.937
Noise annoyance*e* (%)		10.1		10.6		10.2		0.605
**a**Self-reported physician diagnosis. **b**Difference between strata. **c**Based on highest degree of schooling: low, primary school; medium, secondary school; high, technical schools or university. **d**Subjects answering “noise” to the question “Which substances were you working with during the last 3 years?” **e**High noise annoyance corresponds to > 6 on an 11-point scale asking for annoyance by traffic at home when windows are open.								

**Table 2:** Descriptive statistics and Spearman’s correlation coefficients of the environmental exposures used.

Correlation coefficient										
Exposure	Mean exposure	Percentile					Railway noise			Traffic noise			Pollution		
		*n*	10th	50th	90th	Night	Day	Night	Day	PM_10_	NO_2_
Noise [dB(A)]*a*																						
Railway (night)		2,274		16.8 ± 10.9		3		16		30		1.00		—		—		—		—		—
Railway (day)		3,386		18.5 ± 10.6		6		17		38		0.90		1.00		—		—		—		—
Traffic (night)		6,354		38.7 ± 6.9		30		39		47		0.19		0.19		1.00		—		—		—
Traffic (day)		6,354		50.5 ± 7.2		42		50		60		0.19		0.18		0.98		1.00		—		—
Pollution (μg/m^3^)																						
PM_10_		6,412		21.3 ± 7.1		10		21		32		0.37		0.31		0.17		0.16		1.00		—
NO_2_		6,421		23.0 ± 9.9		11		21		37		0.29		0.18		0.29		0.28		0.74		1.00
**a***n* corresponds to participants with estimated nighttime or daytime railway noise.																						

The multivariate regression analysis indicated that a 10 dB(A) increase in railway noise was significantly associated with blood pressure measurements, with stronger associations for nighttime than for daytime railway noise [e.g., model 5: SBP and nighttime noise, β = 0.84; 95% confidence interval (CI): 0.22, 1.46; vs. SBP and daytime noise, β = 0.60; 95% CI: 0.07, 1.13] and for SBP than for DBP (e.g., for nighttime railway noise: SBP, β = 0.84; 95% CI: 0.22, 1.46; DBP, β = 0.44; 95% CI: 0.06, 0.81; Table 3). No significant associations were found for a 10 dB(A) increment of traffic noise in the total study population (Table 3). The stepwise addition of socioeconomic, lifestyle, and health status variables documents their confounding. While adding lifestyle factors to model 1 did not result in a change of effect estimates (e.g., SBP and nighttime railway noise: model 1, β = 0.88; 95% CI: 0.23, 1.54; model 2, β = 0.87; 95% CI: 0.22, 1.51), adding the chronic disease variables yielded a reduction of the effect estimate, albeit with similar confidence intervals (e.g., SBP and nighttime railway noise: model 2, β = 0.87; 95% CI: 0.22, 1.51; model 3, β = 0.79; 95% CI: 0.17, 1.42). The additional adjustment for NO_2_ in the final model resulted in slightly increased effect estimates for both daytime and nighttime railway noise and both SBP and DBP (Table 3). A sensitivity analysis using PM_10_ instead of NO_2_ yielded only marginally different results [final model PM_10_ adjusted, for nighttime railway noise: SBP, β/10 dB(A) = 0.87; 95% CI: 0.24, 1.49; DBP, β/10 dB(A) = 0.44; 95% CI: 0.06, 0.81].

**Table 3:** Association between blood pressure and railway noise by day and night: estimated change of blood pressure (mmHg) for a 10 dB(A) increment of noise.

Night	Day
Model*a*	β/10 dB(A) (95% CI)	*p*-Value	β/10 dB(A) (95% CI)	*p*-Value
Railway noise*b*								
SBP								
Model 4		0.79 (0.17, 1.41)		0.013		0.53 (0.01, 1.07)		0.050
Model 5		0.84 (0.22, 1.46)		0.008		0.60 (0.07, 1.13)		0.028
DBP								
Model 4		0.42 (0.05, 0.79)		0.028		0.18 (–0.14, 0.50)		0.266
Model 5 SBP		0.44 (0.06, 0.81)		0.023		0.21 (–0.11, 0.53)		0.200
Traffic noise*c*								
SBP								
Model 4		–0.01 (–0.60, 0.59)		0.986		–0.11 (–0.68, 0.47)		0.704
Model 5		0.15 (–0.48, 0.77)		0.644		0.05 (–0.56, 0.07)		0.878
DBP								
Model 4		–0.05 (–0.41, 0.30)		0.771		–0.10 (–0.44, 0.24)		0.557
Model 5		–0.15 (–0.36, 0.39)		0.936		–0.04 (–0.40, 0.33)		0.851
**a**Model 4 is corrected for age, age as quadratic polynomial, education, full-time employment status, marital status, study area (random effect), diabetes, mean pulse, antihypertensive medication, self-reported physician-diagnosed hypertension, hearing impairment, and noise exposure at work. Model 5 is corrected for variables of model 4 plus home outdoor estimate NO_2_ exposure. **b**Simultaneously corrected for traffic noise exposure. **c**Simultaneously corrected for railway noise exposure.								

The imputation of the 10% and 25% percentile of the respective exposure in subjects with missing railway exposure data, as well as the exclusion of the subjects with missing values, did not alter the main results (data not shown).

The effect estimates for railway noise were higher among participants who had lived at the same address since the first survey than among those who had moved between surveys, but the corresponding confidence intervals overlapped considerably. The increase in SBP per 10 dB(A) increment of railway noise at night was β = 0.97 (95% CI: 0.04, 1.90) for individuals with stable residence and β = 0.71 (95% CI: –0.10, 1.52) for those who had moved.

Subjects who presumably spent more time at home because of a part-time working status and who were not exposed to occupational noise were defined to be of lower risk of misclassification. Analyses in these subjects yielded an increased effect estimate for blood pressure per 10 dB(A) increment of nighttime railway noise (SBP, β = 1.04; 95% CI: 0.025, 2.05; DBP, β = 0.56; 95% CI: 0.02, 1.11).

The stratified analysis by chronic disease status yielded larger effect estimates in participants reporting physician-diagnosed hypertension, diabetes, and CVD (Table 4). The association of a 10 dB(A) increase in nighttime railway noise with SBP and DBP was significant among participants with hypertension (SBP, β = 2.50; 95% CI: 0.87, 4.11; DBP, β = 1.31; 95% CI: 0.42, 2.22), whereas the corresponding associations for daytime railway noise were not as strong (SBP, β = 1.31; 95% CI: –0.16, 2.78; DBP, β = 0.71; 95% CI: 0.11, 1.53), and no significant association was found in the larger subsample of nonhypertensives. In subjects with self-reported CVD the nighttime railway effect estimates for SBP and DBP per 10 dB(A) were of borderline significance (SBP, β = 2.14; 95% CI: –0.74, 5.0; DBP, β = 1.11; 95% CI: –0.49, 2.7). For participants with and without physician-diagnosed diabetes, railway noise at nighttime was associated with SBP (Table 4). However, the association of a 10 dB(A) increase in railway noise at night with SBP was approximately five times larger among participants with diabetes (SBP, β = 3.7; 95% CI: –0.09, 7.57) than among those without diabetes (β = 0.71; 95% CI: 0.08, 1.34). In contrast, DBP was associated with nighttime railway noise among nondiabetics only. In the subsample of persons with diabetes we also found a significant association between nighttime traffic noise and SBP and borderline significant results for daytime traffic and SBP and DBP, whereas no significant association was found among the paricipants who were not diabetic (Table 4). Similarly, borderline significant associations were seen for nighttime traffic noise among those participants with CVD, but none among those without CVD.

**Table 4:** Association*a* between blood pressure and railway and traffic noise exposure in potentially vulnerable subsamples of the study population.

Participants with no self-reported physician-diagnosed condition					Participants with self-reported physician-diagnosed conditionn				
Condition	*n*	β/10 dB(A) (95% CI)	*p*-Value	*n*	β/10 dB(A) (95% CI)	*p*-Value
Railway noise												
Hypertension		4,494						1,001				
Nighttime												
SBP				0.35 (–0.03, 1.01)		0.305				2.50 (0.87, 4.11)		0.003
DBP				0.19 (–0.21, 0.60)		0.352				1.31 (0.42, 2.22)		0.004
Daytime												
SBP				0.46 (–0.10, 1.02)		0.104				1.31 (–0.16, 2.78)		0.081
DBP				0.11 (–0.23, 0.46)		0.520				0.71 (–0.11, 1.53)		0.088
Diabetes		5,815						179				
Nighttime												
SBP				0.71 (0.08, 1.34)		0.027				3.7 (–0.09, 7.57)		0.056
DBP				0.44 (0.06, 0.82)		0.024				–0.45 (–2.46, 1.55)		0.657
Daytime												
SBP				0.52 (–0.14, 1.05)		0.057				2.33 (–1.15, 5.80)		0.190
DBP				0.24 (–0.89, 0.56)		0.155				–1.0 (–2.78, 0.77)		0.269
CVD*b*		5,533						460				
Nighttime												
SBP				0.68 (0.05, 1.31)		0.035				2.14 (–0.74, 5.0)		0.145
DBP				0.36 (–0.02, 0.75)		0.063				1.11 (–0.49, 2.7)		0.175
Daytime												
SBP				0.55 (0.01, 1.10)		0.045				1.34 (–1.17, 3.85)		0.294
DBP				0.19 (–0.14, 0.52)		0.261				0.84 (–0.57, 2.24)		0.243
Traffic noise												
Hypertension		4,494						1,001				
Nighttime												
SBP				0.150 (–0.49, 0.80)		0.641				0.513 (–1.44, 2.50)		0.608
DBP				0.032 (–0.36, 0.43)		0.872				0.071 (–1.01, 1.16)		0.899
Daytime												
SBP				0.018 (–0.60, 0.63)		0.955				0.674 (–1.27, 2.62)		0.497
DBP				–0.002 (–0.38, 0.38)		0.991				0.013 (–1.07, 1.09)		0.981
Diabetes		5,815						179				
Nighttime												
SBP				0.07 (–0.56, 0.70)		0.832				5.21 (–0.08, 10.50)		0.054
DBP				–0.03 (–0.41, 0.35)		0.875				1.69 (–1.02, 4.40)		0.222
Daytime												
SBP				–0.04 (–0.64, 0.57)		0.909				5.20 (1.35, 10.25)		0.044
DBP				–0.09 (–0.45,0.28)		0.642				2.26 (–0.33, 4.85)		0.088
CVD*b*		5,533						460				
Nighttime												
SBP				–0.07 (–0.57, 0.7)		0.826				2.00 (–0.76, 4.80)		0.155
DBP				–0.06 (–0.45, 0.32)		0.752				0.13 (0.17, 2.85)		0.083
Daytime												
SBP				–0.03 (–0.65, 0.59)		0.922				2.10 (–0.59, 4.79)		0.126
DBP				–0.11 (–0.48, 0.27)		0.575				1.22 (–0.25, 2.69)		0.104
**a**Using model 5 adjusted for age, age as quadratic polynomial, full-time employment status, education, marital status, smoking, alcohol use, BMI, cholesterol, diabetes, mean pulse, hearing impairment, noise exposure at work, home outdoor estimate of NO_2_, equals estimate of NO_2 _study area (random effect), and simultaneously adjusted for other noise exposure (traffic or railway).** b**CVD defined as reported myocardial infarction or stroke.												

## Discussion

Our analysis yielded significant positive associations of railway noise during day and night with SBP and DBP in an adult cohort in Switzerland. Associations were particularly strong among subjects with reported physician-diagnosed hypertension, diabetes, or CVD. In the absence of comparable studies on railway noise, this result is a novel finding. For traffic noise, no association with blood pressure was found in the study population as a whole, whereas stratified analyses yielded a significant positive association with blood pressure in participants with doctor-diagnosed diabetes.

This finding was unexpected since more recent studies have indicated an impact of traffic noise exposure on hypertension in general populations (Belojevic et al. 2008; Chang et al. 2009; de Kluizenaar et al. 2007; Jarup et al. 2008). Possibly because the traffic noise pollution levels in Switzerland are comparably low (Dratva et al. 2010) and the exposure contrast is weaker, the adverse impact of traffic noise could be seen only in a more susceptible subsample. Misclassification of noise exposure might provide another explanation.

Potential misclassification is certainly a limitation of the study. Although we found railway noise to be significantly associated with blood pressure, the data set was missing a considerable number of railway estimates. Various approaches to treat this situation were applied but did not alter the main results. The number of missing traffic noise values was negligible. Also, the SAPALDIA 2 survey did not include information on the location of bedrooms or rooms frequented during daytime relative to the noise source, which could have led to an overestimation of the exposure. Second, the noise data used have been modeled for large-scale exposure mapping, and precision at the household level is lower in low and high exposure categories. The FOEN is currently refining its models, and more precise exposure data will be available for a validation of the results from the next SAPALDIA follow-up assessment currently being conducted (SAPALDIA 3, 2010–2011).

Railway noise estimates are reported to be more valid with less standard uncertainty compared with traffic estimates (BAFU 2009b). Railway and traffic noise differ also in frequency, slope of rise, and absolute levels of noise. Traffic noise from heavily frequented streets corresponds to a continuous exposure without much variation in noise exposure levels, whereas railway noise is characterized by a discontinuous pattern of intermittent very high maximum sound pressure levels and steep slopes of rise. The currently used measure of noise [equivalent sound level (Leq)], based on the average sound level, therefore potentially underestimates the detrimental effect of rail and aircraft noise (Lercher et al. 2010). The intermittent pattern of noise exposure is thought to be more disruptive (Griefahn et al. 2008). Especially during nighttime railway noise causes repeated sleep disturbances and activation of the sympathetic nervous system, which may reduce the normal nocturnal blood pressure (Babisch et al. 2001; Griefahn et al. 2008; Haralabidis et al. 2010), and there is evidence that cardiac responses do not habituate to nighttime noise (Griefahn et al. 2008). However, few studies have addressed railway noise and cardiovascular health outcomes (Babisch 2006; Barregard et al. 2009). This may be due to the smaller percentage of population exposed and the view that railway noise is less aggressive and altogether causes less annoyance (Guski 1999). Compared with aircraft noise exposure, for which an association with increased blood pressure has been shown in the HYENA (Hypertension and Exposure to Noise near Airports) study (Jarup et al. 2008), among others (Babisch and Kamp 2009; Eriksson et al. 2007), railway noise has a similar intermittent and disruptive pattern, although it is much lower in A-weighted decibels than is aircraft noise. Therefore, it may not be surprising to find a positive association with blood pressure for railway noise. The estimated increase in blood pressure associated with a 10-dB(A) increase in railway noise found in our study may fall into the normal homeostasis within a single individual, but on a population level such a shift could lead to substantial increases in CVD. The difference in effect estimates for SBP and DBP may essentially reflect the smaller range and variance of DBP compared with SBP and not different strengths of effects.

The results imply a larger health effect by nighttime noise exposure, which is consistent with results from the HYENA study (Haralabidis et al. 2008) and those from a study of children (Belejovic et al. 2008). Sleep disturbance and activation of the sympathetic nervous system (Griefahn et al. 2008), as well as higher catecholamine levels after nighttime noise exposure (Ising and Kruppa 2004), are possible explanations. The stronger association might also be due to less misclassification of nighttime exposure compared with daytime exposure, which is more likely to be affected by time spent at work or workplace exposure. In both cases we would expect a nondifferential misclassification and thereby an underestimation of the effect. This speculation is supported by our analyses in subjects with presumed lower risk of misclassification, yielding higher effect estimates than in subjects with potentially higher degree of misclassification.

Associations between environmental noise and cardiovascular outcomes may be confounded by concurrent air pollution exposure. Evidence of an effect of air pollution on blood pressure is increasing (Brook et al. 2010). Only a few studies have addressed confounding by air pollution. De Kluizenaar et al. (2007) found no significant association of traffic noise with hypertension, and Beelen et al. (2009) found weaker associations with traffic noise after adjusting for air pollution. The additional adjustment for home outdoor exposure estimates of NO_2_ or PM_10_ in our model did not result in a lower effect estimate either for traffic or railway noise. However, railway-specific particles resulting from the resuspension of particles derived from abrasion of rails or dust might differ in biological properties and be a more important confounder than NO_2_ or traffic PM_10_, which largely reflects traffic and combustion derived pollution. To disentangle these source-specific exposures, one will need reliable models of both exposures. With the pending improvements in noise modeling of FOEN and expansions of source-specific modeling of PM as part of SAPALDIA 3, this issue may be addressed in more detail. Another confounding factor difficult to disentangle from the noise effect is vibration elicited by railway as well as by large vehicles. To our knowledge, studies on health effects of vibration have mainly focused on occupational exposures, so far.

A strength of the presented data is the detailed information on potential predictors and confounders, such as lifestyle and health status, enabling effective control for confounding and specific analyses in potentially more vulnerable subgroups of the population, such as participants with physician-diagnosed hypertension, CVD, or diabetes. The analyses identified subjects reporting physician-diagnosed hypertension as a subpopulation that may be more vulnerable to effects of railway noise on blood pressure. Similarly, stronger associations were seen among participants with a history of CVD, and nighttime railway exposure was more strongly associated with SBP among persons with diabetes than among persons without diabetes. Although associations between blood pressure and traffic noise were weak in the entire sample, stronger associations were estimated for persons with diabetes and for particiants with CVD. This supports our argument that the levels of traffic-noise exposure might have been too low to show an effect in the general population. It is generally accepted that subjects with preexisting chronic disease are more susceptible to external stressors. For example, Babisch (2003) found that the subjects who suffered from chronic disease showed higher noise annoyance and disturbance than did the participants who did not have a chronic disease. A simple explanation is that these subjects lacked the reserve to cope with additional stressors. It could also be that the same physiological pathways are activated and potentiated (Babisch 2003). They also found a higher risk, although nonsignificant, of ischemic heart disease among noise-exposed subjects with preexisting chronic disease compared with healthy subjects (Babisch 2003). Our data support the hypothesis of higher vulnerability to environmental exposures for persons with chronic disease.

## Conclusion

We present evidence of an adverse effect of railway noise on blood pressure, especially for nighttime exposure. Adjustment for estimated home outdoor NO_2_ or PM_10_ exposure did not alter the results, but further investigations into potential confounding by source-specific air pollution are warranted. Effects from traffic noise were not seen in the study population as a whole, but positive associations were estimated in subjects with self-reported diabetes and CVD. The results underline the need to investigate potentially vulnerable populations, because we saw considerably higher effects of both railway and traffic noise exposure among participants with physician-diagnosed hypertension, CVD, and diabetes.
